# Can we predict who will complete outpatient therapy for anorexia nervosa?

**DOI:** 10.1186/2050-2974-3-S1-O42

**Published:** 2015-11-23

**Authors:** Jennifer Jordan, Virginia McIntosh, Frances Carter, Peter Joyce, Cynthia Bulik, Suzanne Luty, Janice McKenzie, Christopher Frampton, Janet Carter

**Affiliations:** 1 University of Otago, New Zealand; 2 Canterbury District Health Board, Christchurch, New Zealand; 3 University of North Carolina at Chapel Hill, USA; 4 The Karolinka Institute, Sweden; 5 University of Canterbury, New Zealand

## 

The literature on characteristics associated with premature termination of treatment (PTT) is beset with conflicting findings.

## Aim

This study examines potential clinical and therapy related predictors in relation to treatment completion status in a randomised psychotherapy trial for anorexia nervosa (AN).

## Methods

Participants were 56 women aged 17-40 years with strict or lenient AN. Treatment completion was defined as completing 15/20 planned sessions. Measures were demographic and clinical variables (eating disorder history, comorbidity and personality measures), patient-rating on the Treatment Credibility scale and early therapy alliance - audiotaped sessions were rated by independent raters using the Vanderbilt Therapeutic Alliance scale (VTAS) and the Vanderbilt Psychotherapy Process Scale (VPPS). Statistics were univariate tests, correlations and logistic regressions.

## Results

Lower self-transcendence scores on the Temperament and Character Inventory, lower treatment credibility ratings and lower therapy alliance on some subscales were associated with PTT.

## Conclusions

Paying close attention to specific patient personality characteristics, patient views of therapy and early process indicators may assist clinicians to retain more patients in treatment and thereby enhance treatment outcomes.

